# Bis(3,5-dimethyl-1*H*-pyrazole-κ*N* ^2^)silver(I) hexa­fluorido­anti­monate

**DOI:** 10.1107/S1600536811010567

**Published:** 2011-03-26

**Authors:** Devyn Crawford, Anthony K. Hofer, Kate L. Edler, Gregory M. Ferrence

**Affiliations:** a CB 4160, Department of Chemistry, Illinois State University, Normal, IL 61790, USA

## Abstract

The title compound, [Ag(C_5_H_8_N_2_)_2_]SbF_6_, contains an Ag^+^ cation almost linearly bonded to two N atoms of dimethylpyrazole ligands [N—Ag—N = 176.54 (18)°]. The structure exhibits hydrogen bonding between the two dimethyl­pyrazole H atoms and two F atoms of one hexa­fluorido­anti­monate anion. Three relatively short Ag⋯F contacts [2.869 (6), 2.920 (7), and 3.094 (7) Å] exist between the cation and three different SbF_6_^−^ anions. The crystal used for data collection was found to be twinned by non-merohedry, with the two components being related by a 180° rotation around the real or reciprocal *a* axis. Integration resulted in 11.2% of the total peaks being assigned to component 1, 11.2% to component 2, and 77.6% to both components.

## Related literature

For related structures and background, see: Gallego *et al.* (2004 ▶, 2005 ▶); Garcia-Pacios *et al.* (2009 ▶); Mohamed & Fackler (2002 ▶). For crystallographic analysis, see: Bruno *et al.* (2004 ▶); Bruker (2005 ▶).
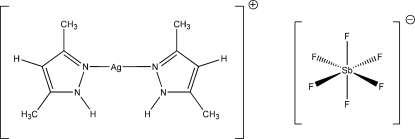


## Experimental

### 

#### Crystal data

[Ag(C_5_H_8_N_2_)_2_]SbF_6_
*M* *_r_* = 535.89
Monoclinic, 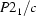

*a* = 7.0242 (7) Å
*b* = 10.9849 (11) Å
*c* = 21.391 (2) Å
β = 91.560 (2)°
*V* = 1649.9 (3) Å^3^
*Z* = 4
Mo *K*α radiation
μ = 2.88 mm^−1^
*T* = 100 K
0.45 × 0.30 × 0.30 mm

#### Data collection

Bruker SMART APEX CCD diffractometer
Absorption correction: multi-scan (*TWINABS*; Bruker, 2008 ▶) *T*_min_ = 0.564, *T*_max_ = 0.746
16455 measured reflections
4913 independent reflections
4686 reflections with *I* > 2σ(*I*)
*R*_int_ = 0.039

#### Refinement

*R*[*F* ^2^ > 2σ(*F* ^2^)] = 0.042
*wR*(*F* ^2^) = 0.138
*S* = 1.20
4913 reflections
204 parameters
H-atom parameters constrained
Δρ_max_ = 1.33 e Å^−3^
Δρ_min_ = −1.47 e Å^−3^

### 

Data collection: *APEX2* (Bruker, 2008 ▶); cell refinement: *SAINT* (Bruker, 2008 ▶); data reduction: *SAINT*; program(s) used to solve structure: *SIR2004* (Burla *et al.*, 2005 ▶); program(s) used to refine structure: *SHELXL97* (Sheldrick, 2008 ▶); molecular graphics: *ORTEP-3 for Windows* (Farrugia, 1997 ▶); software used to prepare material for publication: *WinGX* (Farrugia, 1999 ▶), *publCIF* (Westrip, 2010 ▶) and *Mercury* (Macrae *et al.*, 2006 ▶).

## Supplementary Material

Crystal structure: contains datablocks global, I. DOI: 10.1107/S1600536811010567/fj2400sup1.cif

Structure factors: contains datablocks I. DOI: 10.1107/S1600536811010567/fj2400Isup2.hkl

Additional supplementary materials:  crystallographic information; 3D view; checkCIF report

**Fig. 2.**
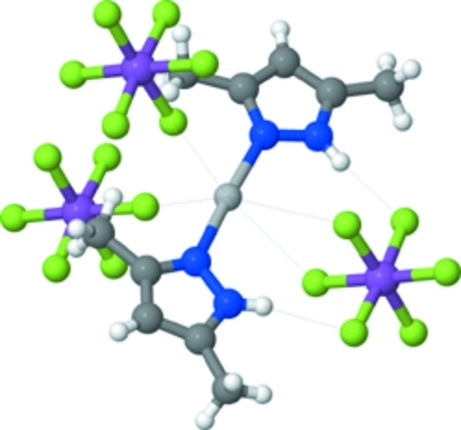
Static view of the enhanced Jmol figure, depicting one silver cation viewed with the three antimonate anions within less than van der Waals' radii. Online enhanced figure can be toggled to show the silver cation surrounded by all antimonate anions within short contact range or the antimonate anion surrounded by all silver cations within short contact range.

Enhanced figure: interactive version of Fig. 2

## Figures and Tables

**Table 1:** Hydrogen-bond geometry (Å, °)

*D*—H⋯*A*	*D*—H	H⋯*A*	*D*⋯*A*	*D*—H⋯*A*
N3—H3⋯F18	0.86	2.16	3.012 (6)	172
N10—H10⋯F17	0.86	2.31	3.149 (7)	167

**Fig. 1.**
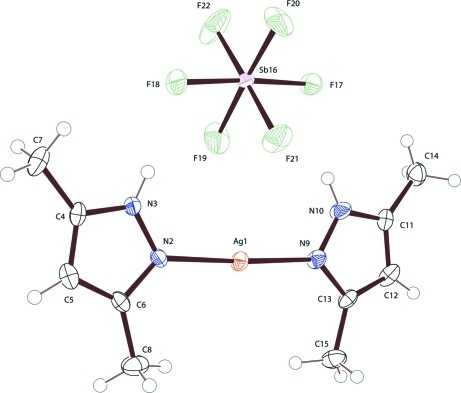
The molecular structure of the title compound with the atomic numbering scheme. Displacement ellipsoids are drawn at the 50% probability level and H-atoms are shown as spheres of arbitrary size.

**Fig. 3.**
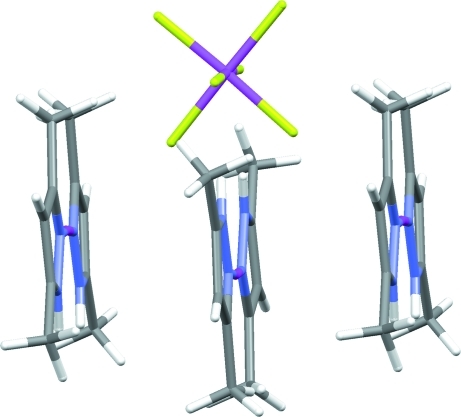
One antimonate anion viewed with all of the silver cations within less than van der Waals' radii.

## supplementary crystallographic information

### Comment

Dimethylnitropyrazolesilver(I) (Gallego *et al.*, 2004; Gallego *et al.*, 2005) and dimethylpyrazolesilver(I) (Mohamed & Fackler, 2002; Garcia-Pacios *et al.*, 2009) complexes have become compounds of interest in recent years. A focus of this research has been on the structural and electronic factors that affect the various interactions of the pyrazole ligand. These bis substituted silver(I) complexes have been observed to most commonly form hydrogen bonds with other solvent anions (Mohamed & Fackler, 2002; Gallego *et al.*, 2004; Gallego *et al.*, 2005).

In the title compound (shown in Figure 1), both hydrogen atoms on the two dimethylpyrazole ligands are H-bonded to one hexafluoridoantimonate anion (distances of 2.158 (4)Å and 2.306 (4) Å). These two fluorine atoms of the anion are displaced slightly toward the hydrogen atoms resulting in a 176.5 (2)° F—Sb—F bond angle. Similar length H-bonds are seen in other dimethylpyrazolesilver(I) and pyrazolesilver(I) complexes. In a similar structure published by Gallego *et al.* (2004), the 3,5-dimethylpyrazole contains an additional nitro group at the pyrazole 4- position. The anion in this structure is CF_3_SO_3_^-1^. In this structure, the anion H-bonds to both pyrazole ligands, however, in this case, it is the silver cation that is structurally strained into an angle of 163.7°. Structures published by Mohamed & Fackler (2002) and Gallego *et al.* (2005) contained pyrazole ligands that did not have the H-atom in a *syn* planar position; however, in these structures each H-atom bonded to a different anion.

A *Mogul* geometry check (Bruno *et al.*, 2004) of the title compound indicates that there are three unusual bond lengths: Ag1—N2 (2.100 Å), Ag1—N9 (2.102 Å), and N10—N9 (1.370 Å). The mean values are 2.139 (18) Å and 1.361 (4) Å, respectively. The bond lengths of these corresponding atoms in a structure published by Garcia-Pacios *et al.* (2009) (a structure that has an identical cation) are 2.087 Å, 2.098 Å, and 1.345Å (all flagged as unusual).

The silver cation is covalently coordinated to two pyrazole ligands. One antimonate anion H-bonds to both of these pyrazole ligands. This antimonate ion together with two additional antimonate ions form three relatively short Ag···F contacts. There is a 2.869 (6) Å separation between Ag1 and F21 of the anion at -*x*,-*y*,-*z*. There is a 2.920 (7) Å separation between Ag1 and F19 of the anion at 1 - *x*,-*y*,-*z*, and there is a 3.094 (7) Å separation between Ag1 and F21 of the anion at *x*,*y*,*z*. A long 3.219 (7) Å A g1···F19 separation effectively places the silver ion in an octahedral coordination environment. The view containing these contacts is shown in the enhanced Jmol figure, Figure 2. Conversely, one antimonate anion is surrounded by three silver cations with which it makes close contacts, shown in Figure 3.

### Experimental

All experimental procedures were conducted in an inert atmosphere. The title compound was prepared by dissolving 0.101 g (0.293 mmol) AgSbF_6_ in 10 ml anhydrous THF. A second solution was prepared separately by dissolving 0.109 g (1.14 mmol) HPz^Me2^ in 50 ml anhydrous THF. The two solutions were combined in a round bottom flask, capped, covered with foil, and stirred for 24 h. Crystals were obtained by decanting the solution into an Erlenmeyer flask and allowing the crystals to form out of the THF solvent *via* slow evaporation.

### Refinement

The crystal under investigation was found to be non-merohedrally twinned. The orientation matrices for the two components were identified using the program Cell Now (Bruker, 2005), with the two components being related by a 180 degree rotation around the real or reciprocal axis *a*. The two components were integrated using *SAINT*, resulting in a total of 18534 reflections. 2075 reflections (1041 unique) involved component 1 only (mean I/σ = 20.7), 2079 reflections (1040 unique) involved component 2 only (mean I/σ = 20.2), and 14380 reflections (4690 unique) involved both components (mean I/σ = 16.7). The exact twin matrix identified by the integration program was found to be (1.000 - 0.001 0.000 / -0.003 - 1.000 0.000 / -0.162 0.000 - 1.000).

The data were corrected for absorption using twinabs, and the structure was solved using direct methods with only the non-overlapping reflections of component 1. The structure was refined using the hklf 5 routine with all reflections of component 1 (including the overlapping ones), resulting in a BASF value of 0.45154.

The *R*_int_ value given is for all reflections and is based on agreement between observed single and composite intensities and those calculated from refined unique intensities and twin fractions (TWINABS; Bruker, 2008).

All non-H atoms were refined anisotropically. All H atoms were initially identified through difference Fourier syntheses then removed and included in the refinement in the riding-model approximation (C–H = 0.93 and 0.96Å for Ar–H and CH_3_; N–H = 0.86 Å; *U*_iso_(H) = 1.2Ueq(C) except for methyl groups, where *U*_iso_(H) = 1.5Ueq(C)).

### Crystal data

**Table:**

[Ag(C_5_H_8_N_2_)_2_]SbF_6_	*F*(000) = 1024
*M**_r_* = 535.89	*D*_x_ = 2.157 Mg m^−^^3^
Monoclinic, *P*2_1_/*c*	Mo *K*α radiation, λ = 0.71073 Å
Hall symbol: -P 2ybc	Cell parameters from 18535 reflections
*a* = 7.0242 (7) Å	θ = 2.7–31.2°
*b* = 10.9849 (11) Å	µ = 2.88 mm^−^^1^
*c* = 21.391 (2) Å	*T* = 100 K
β = 91.560 (2)°	Rod, colourless
*V* = 1649.9 (3) Å^3^	0.45 × 0.3 × 0.3 mm
*Z* = 4	

### Data collection

**Table:**

Bruker SMART APEX CCD diffractometer	4686 reflections with *I* > 2σ(*I*)
ω scans	*R*_int_ = 0.039
Absorption correction: multi-scan (TWINABS; Bruker, 2008)	θ_max_ = 31.6°, θ_min_ = 1.9°
*T*_min_ = 0.564, *T*_max_ = 0.746	*h* = −10→10
16455 measured reflections	*k* = 0→15
4913 independent reflections	*l* = 0→31

### Refinement

**Table:**

Refinement on *F*^2^	0 restraints
Least-squares matrix: full	H-atom parameters constrained
*R*[*F*^2^ > 2σ(*F*^2^)] = 0.042	*w* = 1/[σ^2^(*F*_o_^2^) + (0.0685*P*)^2^ + 9.0304*P*] where *P* = (*F*_o_^2^ + 2*F*_c_^2^)/3
*wR*(*F*^2^) = 0.138	(Δ/σ)_max_ < 0.001
*S* = 1.20	Δρ_max_ = 1.33 e Å^−^^3^
4913 reflections	Δρ_min_ = −1.47 e Å^−^^3^
204 parameters	

### Special details

**Table:**

Geometry. All e.s.d.'s (except the e.s.d. in the dihedral angle between two l.s. planes) are estimated using the full covariance matrix. The cell e.s.d.'s are taken into account individually in the estimation of e.s.d.'s in distances, angles and torsion angles; correlations between e.s.d.'s in cell parameters are only used when they are defined by crystal symmetry. An approximate (isotropic) treatment of cell e.s.d.'s is used for estimating e.s.d.'s involving l.s. planes.

### Fractional atomic coordinates and isotropic or equivalent isotropic displacement parameters (Å^2^)

**Table:**

	*x*	*y*	*z*	*U*_iso_*/*U*_eq_	
Ag1	0.24944 (8)	−0.03039 (4)	−0.018995 (19)	0.02105 (12)	
N2	0.2337 (9)	0.0994 (4)	−0.0910 (2)	0.0186 (9)	
N3	0.2235 (8)	0.2210 (5)	−0.0802 (2)	0.0193 (9)	
H3	0.2263	0.2529	−0.0434	0.023*	
C4	0.2085 (10)	0.2865 (6)	−0.1338 (3)	0.0222 (12)	
C5	0.2061 (10)	0.2033 (6)	−0.1820 (3)	0.0246 (12)	
H5	0.1948	0.2203	−0.2245	0.029*	
C6	0.2238 (10)	0.0878 (6)	−0.1544 (3)	0.0197 (11)	
C7	0.1939 (13)	0.4221 (7)	−0.1335 (4)	0.0343 (17)	
H7A	0.1171	0.4476	−0.0995	0.051*	
H7B	0.1364	0.4492	−0.1723	0.051*	
H7C	0.3189	0.4568	−0.1287	0.051*	
C8	0.2292 (12)	−0.0346 (6)	−0.1844 (3)	0.0319 (14)	
H8A	0.2809	−0.0928	−0.1551	0.048*	
H8B	0.3078	−0.0313	−0.2203	0.048*	
H8C	0.1025	−0.0584	−0.197	0.048*	
N9	0.2625 (9)	−0.1517 (4)	0.0570 (2)	0.0213 (10)	
N10	0.2780 (11)	−0.1124 (5)	0.1177 (2)	0.0278 (12)	
H10	0.2789	−0.0369	0.1284	0.033*	
C11	0.2915 (10)	−0.2048 (6)	0.1585 (3)	0.0229 (12)	
C12	0.3016 (10)	−0.3100 (6)	0.1232 (3)	0.0247 (13)	
H12	0.3213	−0.3887	0.138	0.03*	
C13	0.2760 (9)	−0.2738 (5)	0.0606 (3)	0.0200 (11)	
C14	0.3053 (12)	−0.1818 (7)	0.2275 (3)	0.0304 (15)	
H14A	0.2692	−0.0992	0.2359	0.046*	
H14B	0.434	−0.1951	0.2422	0.046*	
H14C	0.2217	−0.2363	0.2486	0.046*	
C15	0.2704 (12)	−0.3504 (6)	0.0031 (3)	0.0291 (14)	
H15A	0.3054	−0.3019	−0.0321	0.044*	
H15B	0.1439	−0.3817	−0.0039	0.044*	
H15C	0.3581	−0.4169	0.0081	0.044*	
Sb16	0.25087 (6)	0.25666 (3)	0.113329 (15)	0.01885 (11)	
F17	0.2849 (8)	0.1468 (4)	0.18060 (18)	0.0322 (10)	
F18	0.2194 (9)	0.3560 (4)	0.04231 (18)	0.0349 (11)	
F19	0.4415 (9)	0.1718 (6)	0.0716 (3)	0.0513 (17)	
F20	0.0553 (10)	0.3341 (6)	0.1543 (3)	0.057 (2)	
F21	0.0764 (9)	0.1462 (6)	0.0768 (3)	0.0456 (15)	
F22	0.4263 (11)	0.3651 (6)	0.1477 (4)	0.065 (2)	

### Atomic displacement parameters (Å^2^)

**Table:**

	*U*^11^	*U*^22^	*U*^33^	*U*^12^	*U*^13^	*U*^23^
Ag1	0.0268 (2)	0.01625 (19)	0.02011 (18)	0.0007 (3)	−0.00010 (18)	0.00363 (14)
N2	0.022 (3)	0.020 (2)	0.0143 (17)	0.001 (2)	0.004 (2)	0.0008 (16)
N3	0.023 (3)	0.019 (2)	0.0156 (19)	−0.004 (2)	0.0010 (19)	0.0003 (16)
C4	0.024 (3)	0.021 (3)	0.022 (2)	−0.001 (2)	0.001 (2)	0.009 (2)
C5	0.021 (3)	0.032 (3)	0.020 (2)	0.002 (3)	0.004 (2)	0.006 (2)
C6	0.017 (3)	0.027 (3)	0.015 (2)	0.001 (2)	0.004 (2)	−0.0004 (19)
C7	0.037 (4)	0.021 (3)	0.045 (4)	−0.001 (3)	0.002 (4)	0.012 (3)
C8	0.032 (4)	0.027 (3)	0.037 (3)	0.002 (3)	0.005 (3)	−0.010 (3)
N9	0.026 (3)	0.015 (2)	0.023 (2)	0.003 (2)	−0.003 (2)	0.0037 (17)
N10	0.046 (4)	0.014 (2)	0.023 (2)	0.000 (3)	−0.003 (3)	0.0012 (18)
C11	0.021 (3)	0.022 (3)	0.025 (3)	−0.002 (2)	0.003 (2)	0.006 (2)
C12	0.021 (3)	0.019 (3)	0.034 (3)	−0.003 (2)	−0.014 (3)	0.007 (2)
C13	0.016 (3)	0.014 (2)	0.030 (3)	0.001 (2)	−0.002 (2)	0.001 (2)
C14	0.034 (4)	0.032 (3)	0.025 (3)	−0.004 (3)	−0.001 (3)	0.006 (3)
C15	0.035 (4)	0.024 (3)	0.027 (3)	−0.001 (3)	−0.010 (3)	−0.004 (2)
Sb16	0.0264 (2)	0.01299 (17)	0.01709 (17)	0.0009 (2)	−0.00057 (15)	−0.00089 (11)
F17	0.049 (3)	0.0220 (18)	0.0257 (17)	0.009 (2)	0.000 (2)	0.0061 (14)
F18	0.055 (3)	0.0278 (19)	0.0218 (16)	0.001 (2)	−0.005 (2)	0.0096 (15)
F19	0.045 (3)	0.049 (4)	0.061 (4)	0.019 (3)	0.028 (3)	0.010 (3)
F20	0.080 (4)	0.046 (4)	0.047 (4)	0.040 (3)	0.034 (3)	0.011 (3)
F21	0.054 (3)	0.040 (3)	0.042 (3)	−0.018 (3)	−0.018 (2)	−0.002 (3)
F22	0.099 (5)	0.039 (4)	0.056 (4)	−0.035 (4)	−0.047 (4)	0.015 (3)

### Geometric parameters (Å, °)

**Table:**

Ag1—N2	2.100 (5)	N10—C11	1.341 (8)
Ag1—N9	2.101 (5)	N10—H10	0.86
N2—N3	1.358 (7)	C11—C12	1.383 (10)
N2—C6	1.360 (7)	C11—C14	1.497 (9)
N3—C4	1.354 (7)	C12—C13	1.403 (9)
N3—H3	0.86	C12—H12	0.93
C4—C5	1.377 (9)	C13—C15	1.491 (9)
C4—C7	1.494 (9)	C14—H14A	0.96
C5—C6	1.404 (9)	C14—H14B	0.96
C5—H5	0.93	C14—H14C	0.96
C6—C8	1.490 (9)	C15—H15A	0.96
C7—H7A	0.96	C15—H15B	0.96
C7—H7B	0.96	C15—H15C	0.96
C7—H7C	0.96	Sb16—F22	1.852 (6)
C8—H8A	0.96	Sb16—F20	1.856 (5)
C8—H8B	0.96	Sb16—F19	1.877 (6)
C8—H8C	0.96	Sb16—F18	1.879 (4)
N9—C13	1.348 (7)	Sb16—F21	1.880 (5)
N9—N10	1.371 (7)	Sb16—F17	1.888 (4)
			
N2—Ag1—N9	176.54 (18)	N10—C11—C14	121.0 (6)
N3—N2—C6	105.1 (5)	C12—C11—C14	132.6 (6)
N3—N2—Ag1	123.0 (3)	C11—C12—C13	106.1 (5)
C6—N2—Ag1	131.9 (4)	C11—C12—H12	126.9
C4—N3—N2	112.4 (5)	C13—C12—H12	126.9
C4—N3—H3	123.8	N9—C13—C12	110.1 (5)
N2—N3—H3	123.8	N9—C13—C15	120.9 (5)
N3—C4—C5	106.3 (5)	C12—C13—C15	128.9 (6)
N3—C4—C7	122.0 (6)	C11—C14—H14A	109.5
C5—C4—C7	131.7 (6)	C11—C14—H14B	109.5
C4—C5—C6	106.6 (5)	H14A—C14—H14B	109.5
C4—C5—H5	126.7	C11—C14—H14C	109.5
C6—C5—H5	126.7	H14A—C14—H14C	109.5
N2—C6—C5	109.6 (5)	H14B—C14—H14C	109.5
N2—C6—C8	120.8 (6)	C13—C15—H15A	109.5
C5—C6—C8	129.6 (5)	C13—C15—H15B	109.5
C4—C7—H7A	109.5	H15A—C15—H15B	109.5
C4—C7—H7B	109.5	C13—C15—H15C	109.5
H7A—C7—H7B	109.5	H15A—C15—H15C	109.5
C4—C7—H7C	109.5	H15B—C15—H15C	109.5
H7A—C7—H7C	109.5	F22—Sb16—F20	90.6 (4)
H7B—C7—H7C	109.5	F22—Sb16—F19	91.9 (4)
C6—C8—H8A	109.5	F20—Sb16—F19	177.3 (3)
C6—C8—H8B	109.5	F22—Sb16—F18	90.6 (3)
H8A—C8—H8B	109.5	F20—Sb16—F18	92.5 (3)
C6—C8—H8C	109.5	F19—Sb16—F18	88.5 (3)
H8A—C8—H8C	109.5	F22—Sb16—F21	178.7 (4)
H8B—C8—H8C	109.5	F20—Sb16—F21	90.5 (3)
C13—N9—N10	104.8 (5)	F19—Sb16—F21	87.0 (3)
C13—N9—Ag1	132.7 (4)	F18—Sb16—F21	88.7 (3)
N10—N9—Ag1	122.3 (4)	F22—Sb16—F17	92.2 (3)
C11—N10—N9	112.4 (5)	F20—Sb16—F17	90.7 (3)
C11—N10—H10	123.8	F19—Sb16—F17	88.2 (3)
N9—N10—H10	123.8	F18—Sb16—F17	175.70 (18)
N10—C11—C12	106.3 (5)	F21—Sb16—F17	88.4 (3)
			
C6—N2—N3—C4	−0.3 (8)	C13—N9—N10—C11	−2.9 (9)
Ag1—N2—N3—C4	−178.1 (5)	Ag1—N9—N10—C11	−177.6 (5)
N2—N3—C4—C5	0.9 (8)	N9—N10—C11—C12	5.1 (9)
N2—N3—C4—C7	179.6 (7)	N9—N10—C11—C14	−178.7 (7)
N3—C4—C5—C6	−1.1 (8)	N10—C11—C12—C13	−5.1 (8)
C7—C4—C5—C6	−179.6 (8)	C14—C11—C12—C13	179.3 (8)
N3—N2—C6—C5	−0.4 (8)	N10—N9—C13—C12	−0.6 (8)
Ag1—N2—C6—C5	177.1 (5)	Ag1—N9—C13—C12	173.3 (5)
N3—N2—C6—C8	−179.5 (6)	N10—N9—C13—C15	−178.4 (7)
Ag1—N2—C6—C8	−2.0 (11)	Ag1—N9—C13—C15	−4.5 (11)
C4—C5—C6—N2	1.0 (8)	C11—C12—C13—N9	3.6 (8)
C4—C5—C6—C8	180.0 (7)	C11—C12—C13—C15	−178.8 (7)

### Hydrogen-bond geometry (Å, °)

**Table:**

*D*—H···*A*	*D*—H	H···*A*	*D*···*A*	*D*—H···*A*
N3—H3···F18	0.86	2.16	3.012 (6)	172
N10—H10···F17	0.86	2.31	3.149 (7)	167
